# Prevalence and risk factors of passive suicidal ideation among healthcare professional during residency

**DOI:** 10.1192/j.eurpsy.2025.910

**Published:** 2025-08-26

**Authors:** A. F. Rodríguez Barrera, M. Cabello Salmerón, A. Cano Arenas, M. F. Bravo Ortiz, D. Carracedo Sanchidrián, D. Hernández-Calle, E. Lara Pérez

**Affiliations:** 1 Universidad Autónoma de Madrid; 2 Hospital Universitario La Paz; 3 Hospital Ramón y Cajal; 4 Universidad Complutense de Madrid, Madrid, Spain

## Abstract

**Introduction:**

Health professionals are a vulnerable population, prone to mental health problems such as suicidal behaviors. Suicidal ideation (passive and active) is a potential indicator of committing suicide. Passive suicide ideation has been undervalued as an indicator of suicide risk for not having a plan to commit suicide. Despite the high risk, few studies have examined the prevalence and factors related to the presence of passive suicidal ideation in healthcare residents.

**Objectives:**

Determine the prevalence and associated factors of passive suicidal ideation among resident healthcare professionals, including physicians, nurses, and psychologists.

**Methods:**

This cross-sectional study involved a non-probabilistic sample of 775 healthcare professionals. Data were collected via a self-administered survey distributed to teaching units in hospitals across Spain. Key variables included passive suicidal ideation (yes/no), year of residency, and personality traits (neuroticism, extraversion, openness, agreeableness, conscientiousness), assessed with the Spanish adaptation of the NEO-PI-R Personality Inventory. Descriptive analyses summarized categorical and continuous variables. Associations between passive suicidal ideation and independent variables (age, gender, personality traits, drug consumption, mental health specialization, residency program) were analyzed using logistic regression models.

**Results:**

Among the total sample, 13.42% exhibited passive suicidal ideation. In terms of personality traits, individuals with higher levels of neuroticism and openness demonstrated an increased likelihood of experiencing passive suicidal ideation, whereas higher scores in extraversion were associated with a decreased probability of such ideation. Additionally, the use of prescribed medication and instances of self-medication were found to be related to passive suicidal ideation.

**Conclusions:**

A notable proportion of healthcare professionals experience suicidal ideation during their residency. The findings of this study indicate that preventive measures for suicidal ideation should be implemented during the residency period for healthcare professionals.

**Disclosure of Interest:**

None Declared

